# Comparative analysis of complement C3 and C4 serum levels for outcome prediction in ANCA-associated renal vasculitis

**DOI:** 10.1007/s40620-022-01414-w

**Published:** 2022-08-13

**Authors:** Désirée Tampe, Eva Baier, Samy Hakroush, Björn Tampe

**Affiliations:** 1 Department of Nephrology and Rheumatology, University Medical Center Göttingen, Göttingen, Germany; 2 Institute of Pathology, University Medical Center Göttingen, Göttingen, Germany

**Keywords:** ANCA-associated vasculitis, Complement C3, Complement C4

## Abstract

**Background:**

The activation of the complement system contributes essentially to the pathogenesis of anti-neutrophil cytoplasmic antibody (ANCA)-associated renal vasculitis. We here aimed to directly compare levels of C3 and C4 for outcome prediction in ANCA-associated renal vasculitis.

**Methods:**

Serum levels of complement components C3 and C4 were directly compared in association with clinical and outcome data in a retrospective cohort of ANCA-associated renal vasculitis.

**Results:**

As compared to poor outcome prediction by low levels of complement C3 (*p* = 0.0093), low levels of complement C4 did not associate with early requirement of kidney replacement therapy (KRT) or death (*p* = 0.2396). In the subgroup that experienced KRT or death, low C3 levels identified 11/14 (78.6%, *p* = 0.0071) and C4 levels 9/14 (64.3%, *p* = 0.1786) cases. Interestingly, 2/14 (14.3%) patients that experienced KRT or death had isolated C4 lowering, and combining low C3 and/or C4 levels identified 13/14 (92.3%, *p* < 0.0001) cases in this subgroup. Non-superiority to predict poor outcome by low C3 and/or C4 as compared to C3 alone in the total cohort was attributed to 4/24 (16.7%) patients with isolated C4 lowering in the subgroup that did not experience KRT or death.

**Conclusion:**

While low levels of complement C3 were superior in predicting poor outcome in ANCA-associated renal vasculitis, a minor fraction with poor outcome had isolated C4 lowering not captured by serum C3 measurements. Therefore, detailed knowledge of distinct complement components contributing to kidney injury could be of relevance to improve current strategies targeting the complement system in ANCA-associated renal vasculitis.

**Graphical abstract:**

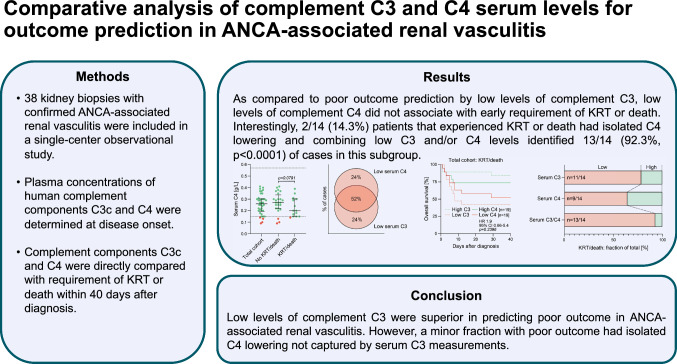

## Introduction

Anti-neutrophil cytoplasmic antibody (ANCA)-associated vasculitis (AAV) is a small vessel vasculitis affecting multiple organ systems, including the kidney. Small vessels in the kidney include small-sized arteries, capillaries, and venules. Disease manifestations in the kidney are common and usually characterized by pauci-immune glomerulonephritis with only minor, if any, immunoglobulin and complement depositions in the vascular system. On a mechanistic level, pathogenic ANCA autoantibodies activate neutrophils, causing a release of inflammatory cytokines, reactive oxygen species, and lytic enzymes, resulting in inflammation and vascular injury. The activation of the complement system contributes essentially to the pathogenesis of ANCA-associated renal vasculitis by autoantibody-antigen recognition directed against host cells [1]. Activation of the complement system induces neutrophil adhesion and formation of neutrophil-platelet aggregates in vascular endothelial cells, implicating a direct mechanistic link between complement activation and vascular injury [1]. The measurement of serum complement C3 and C4 with immunoassays is routinely used in clinical practice to determine and monitor complement activation. Importantly, low serum complement C3 levels without hypocomplementemia per se are an independent predictor of poor renal prognosis in patients with ANCA-associated renal vasculitis [2–5]. By contrast, low serum complement C4 levels alone were not associated with renal outcome [2]. However, serum levels of complement C4 have not been directly compared and analyzed in combination with serum C3 levels. Therefore, we here aimed to directly compare levels of C3 and C4 with regard to its clinical implications in ANCA-associated renal vasculitis.

## Methods

### Study population

A total of 38 cases with ANCA-associated renal vasculitis at the University Medical Center Göttingen were retrospectively included between 2015 and 2020 (Table 1), the patient cohort has previously been described [3, 6–10]. While no formal approval was required for the use of routine clinical data, a favorable ethical opinion was granted by the local Ethics committee (no. 22/2/14 and 28/09/17). At admission, the Birmingham Vasculitis Activity Score (BVAS) version 3 was assessed [11]. Medical records were used to obtain data on age, sex, duration of disease onset before admission, diagnosis (MPA or GPA) and laboratory results. The estimated glomerular filtration rate (eGFR) was calculated using the Chronic Kidney Disease Epidemiology Collaboration (CKD-EPI) equation [12]. Kidney replacement therapy (KRT) was performed intermittently in all cases. Indications for KRT included severe electrolyte and acid–base abnormalities, volume overload and encephalopathy.

**Table 1:** Characteristics of the total cohort of ANCA-associated renal vasculitis

	Value	Normal range
Serum C3–g/L	1.30 (0.99–1.41)	0.82–1.93
Serum C4–g/L	0.26 (0.20–0.30)	0.15–0.57
Serum creatinine–mg/dL	3.16 (1.48–5.22)	0.70–1.20
eGFR–mL/min/1.73 m^2^	17.25 (8.78–47.65)	> 60
CRP–mg/L	60.50 (19.50–101.15)	≤ 5.00
Female sex–no. (%)	17 (44.74)	
Age–years	66.50 (50.75–74.00)	
ANCA diagnosis		
MPA–no. (%)	22 (57.89)	
GPA–no. (%)	16 (42.11)	
ANCA subtype		
MPO–no. (%)	22 (57.89)	
PR3–no. (%)	16 (42.11)	
Relapse–no. (%)	6 (15.79)	
BVAS–points	18 (15.00–20.25)	
KRT within 40 days–no. (%)	13 (34.21)	
Death within 40 days–no. (%)	1 (2.63)	
Berden class		
Sclerotic class–no. (%)	2 (5.26)	
Crescentic class–no. (%)	14 (36.84)	
Focal class–no. (%)	19 (50)	
Mixed class–no. (%)	3 (7.89)	
ANCA Renal Risk Score		
High risk–no. (%)	6 (15.79)	
Intermediate risk–no. (%)	16 (42.11)	
Low risk–no. (%)	16 (42.11)	
Histopathological lesions		
Normal glomeruli–% of total	49.47 (23.37–73.07)	
Crescentic glomeruli–% of total	35.94 (8.17–56.85)	
Necrotic glomeruli–% of total	18.34 (5.77–50.27)	
Sclerotic glomeruli–% of total	2.82 (0.00–21.11)	
IF/TA–%	20.00 (10.00–40.00)	

*ANCA* anti-neutrophil cytoplasmic antibody, *BVAS* Birmingham Vasculitis Activity Score, *CRP* C-reactive protein, *eGFR* estimated glomerular filtration rate (CKD-EPI), *GPA* granulomatosis with polyangiitis, *IF/TA* interstitial fibrosis/tubular atrophy, *IQR* interquartile range, *KRT* kidney replacement therapy, *MPA* microscopic polyangiitis, *MPO* myeloperoxidase, *no.* number, *PR3* proteinase 3                                                                                                                                                                              Continuous variables are expressed as median and IQR, categorical variables are presented as frequency and percentage

### Renal histopathology

A renal pathologist evaluated kidney biopsies and was blinded to data analysis. Periodic acid-Schiff stainings were performed by automated slide stainer Tissue-Tek Prisma (Sakura Finetek Europe, Alphen aan den Rijn, Netherlands) according to the manufacturer’s protocol. Within a kidney biopsy, each glomerulus was scored separately for the presence of necrosis, crescents and global sclerosis. Based on these scores, histopathological subgrouping according to Berden et al. into focal, crescentic, mixed or sclerotic class was performed [13]. Furthermore, the ANCA renal risk score according to Brix et al. into low, medium or high risk was calculated [14].

### C3 and C4 measurements

Plasma concentrations of human complement components C3c (9D9621, Abbott, Chicago, USA) and C4 (9D9721, Abbott, Chicago, USA) were determined by turbidimetric measurements on the ARCHITECT-C module. Normal range plasma concentrations for circulating C3c is defined between 0.82–1.93 g/L and C4 between 0.15–0.57 g/L.

### Statistical methods

Variables were tested for normal distribution using the Shapiro–Wilk test. Statistical comparisons were not formally powered or prespecified. Non-normally distributed continuous variables are shown as median and interquartile range (IQR), categorical variables are presented as frequency and percentage. For group comparisons, the Mann–Whitney *U*-test was used to determine differences in medians. Non-parametric between-group-comparisons were performed with Pearson’s Chi-square test. Serum levels of complement C3 and C4 were directly compared by simple regression analysis. Survival-curve analyses were performed using the Kaplan–Meier method, comparison of survival curves was performed with log rank (Mantel-Cox) testing. Data analyses were performed with GraphPad Prism (version 8.4.3 for MacOS, GraphPad Software, San Diego, California, USA). A probability (*p*) value of < 0.05 was considered statistically significant.

## Results

**Fig. 1:**
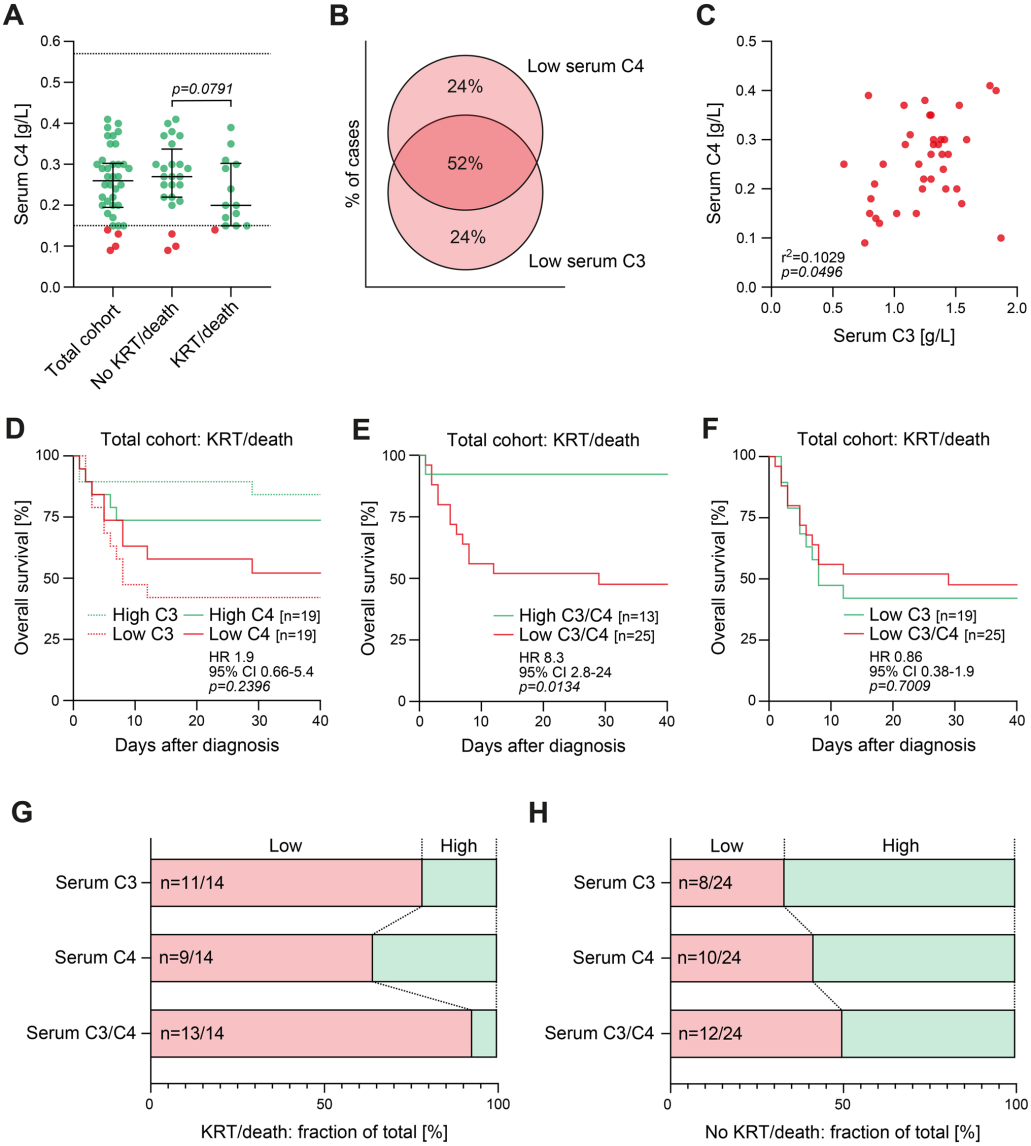
Comparative analysis of complement C3 and C4 serum levels for outcome prediction in ANCA-associated renal vasculitis. **A** Serum levels of complement C4 in the total cohort and according to requirement of KRT or death during follow-up. The scatter dot plots include median ± IQR compared by one-tailed Mann–Whitney test, the dotted lines represent upper and lower normal range of serum C4 levels in our institution. **B** Among 25/38 (65.8%) with low serum levels of C3 and/or C4, distribution of low C3 and C4 levels are shown. **C** Correlation between serum levels of complement C3 and C4 are shown. Values were directly compared by simple regression analysis. **D**, **F** Overall survival (KRT/death) within 40 days after diagnosis according to low or high levels of serum C3, C4, or combined C3 and/or C4. Comparison of survival curves was performed with log rank (Mantel-Cox) testing. **G** In the subgroup that experienced KRT or death, fraction of total is shown according to serum levels of C3, C4, or combined C3 and/or C4. **H** In the subgroup that did not experience KRT or death, fraction of total is shown according to serum levels of C3, C4, or combined C3 and/or C4. *ANCA* anti-neutrophil cytoplasmic antibody, *HR* hazard ratio, *IQR* interquartile range, *KRT* kidney replacement therapy

While only 4/38 (10.5%) patients had hypocomplementemia with C4 levels below the normal range (< 0.15 g/L), patients who required KRT (13/38) or died (1/38) tended to have lower C4 (*p* = 0.0791, Fig. 1A). Low complement C4 below median serum levels (< 0.26 g/L) was only associated with older age, whereas kidney function, ANCA subtype, relapsing disease, systemic vasculitis activity, or classification of ANCA-associated renal vasculitis did not differ (Table 2). By contrast, low complement C3 below median levels (< 1.3 g/L) correlated with kidney injury requiring KRT within a follow-up of 40 days after diagnosis (*p* = 0.0167), not associated with any histopathological lesion or subtyping of ANCA-associated renal vasculitis (Table 3). Among 25/38 (65.8%) with low serum levels of C3 and/or C4, most cases of ANCA-associated renal vasculitis showed an overlap of low C3 and C4 levels (52%), and there were subsets of patients with isolated C4 lowering (24%, Fig. 1B). Direct comparison of serum complement C3 and C4 levels confirmed a positive association with each other (Fig. 1C). As compared to previously described prediction of poor outcome by low levels of complement C3 (*p* = 0.0093), low levels of complement C4 did not associate with early requirement of KRT or death within a follow-up of 40 days after diagnosis (*p* = 0.2396, Fig. 1D) [3]. Combined C3 and/or C4 effectively predicted poor outcome (*p* = 0.0134, Fig. 1E), while direct comparison revealed no superiority of low C3 and/or C4 as compared to low complement levels of C3 alone (Fig. 1F). In the subgroup that experienced KRT or death, low serum complement C3 levels identified 11/14 (78.6%, *p* = 0.0071) and complement C4 levels 9/14 (64.3%, *p* = 0.1786) cases (Fig. 1G). Interestingly, 2/14 (14.3%) patients that experienced KRT or death had isolated C4 lowering, and combining low serum levels of C3 and/or C4 identified 13/14 (92.3%, *p* < 0.0001) cases in this subgroup (Fig. 1G). Non-superiority to predict poor outcome by low C3 and/or C4 as compared to low complement levels of C3 alone in the total cohort was attributed to a subset of 4/24 (16.7%) patients with isolated C4 lowering in the subgroup that did not experience KRT or death (Fig. 1H). In summary, this comparative analysis revealed superiority in predicting poor outcome by low levels of complement C3 as compared to C4 in ANCA-associated renal vasculitis. However, a minor fraction that experienced KRT or death had isolated C4 lowering not captured by serum C3 measurements.

**Table 2:** Group comparison according to median serum levels of complement C4

	C4 < 0.26 g/L	C4 ≥ 0.26 g/L	*p* value
Serum C3–g/L	1.18 (0.84–1.40)	1.32 (1.25–1.44)	0.0313
Serum C4–g/L	0.20 (0.15–0.22)	0.30 (0.29–0.37)	< 0.0001
Serum creatinine–mg/dL	3.14 (1.49–5.26)	3.17 (1.12–4.93)	0.7347
eGFR–mL/min/1.73 m^2^	17.60 (8.40–35.90)	15.80 (9.60–80.60)	0.5584
CRP–mg/L	57.40 (16.40–104.00)	63.60 (19.90–100.20)	0.8567
Female sex–no. (%)	10 (52.63)	7 (36.84)	0.3277
Age–years	69 (61–76)	55 (49–70)	0.0105
ANCA diagnosis			
MPA–no. (%)	10 (52.63)	12 (63.16)	
GPA–no. (%)	9 (47.37)	7 (36.84)	0.5111
ANCA subtype			
MPO–no. (%)	9 (47.37)	13 (68.42)	
PR3–no. (%)	10 (52.63)	6 (31.58)	0.1888
Relapse–no. (%)	4 (21.05)	2 (10.53)	0.3736
BVAS–points	18 (15.00–20.00)	18 (15–21)	0.9937
KRT within 40 days–no. (%)	8 (42.11)	5 (26.32)	0.3050
Death within 40 days–no. (%)	1 (5.26)	0 (0.00)	0.3109
Berden class			
Sclerotic class–no. (%)	1 (5.26)	1 (5.26)	
Crescentic class–no. (%)	8 (42.11)	6 (31.58)	
Focal class–no. (%)	9 (47.37)	10 (52.63)	
Mixed class–no. (%)	1 (5.26)	2 (10.53)	0.8798
ANCA Renal Risk Score			
High risk–no. (%)	3 (15.79)	3 (15.79)	
Intermediate risk–no. (%)	9 (47.37)	7 (36.84)	
Low risk–no. (%)	7 (36.84)	9 (47.37)	0.7788
Histopathological lesions			
Normal glomeruli–% of total	48.94 (24.49–70.59)	54.35 (20.00–83.33)	0.8455
Crescentic glomeruli–% of total	37.50 (10.00–55.56)	33.33 (0.00–60.71)	0.5283
Necrotic glomeruli–% of total	12.50 (0.00–55.56)	28.00 (9.09–46.67)	0.4359
Sclerotic glomeruli–% of total	9.09 (0.00–21.28)	0.00 (0.00–18.18)	0.3379
IF/TA–%	20.00 (10.00–40.00)	20.00 (5.00–40.00)	0.9361

*ANCA* anti-neutrophil cytoplasmic antibody, *BVAS* Birmingham Vasculitis Activity Score, *CRP* C-reactive protein, *eGFR* estimated glomerular filtration rate (CKD-EPI), *GPA* granulomatosis with polyangiitis, *IF/TA* interstitial fibrosis/tubular atrophy, *IQR* interquartile range, *KRT* kidney replacement therapy, *MPA* microscopic polyangiitis, *MPO* myeloperoxidase, *no.* number, *PR3* proteinase 3 Continuous variables are expressed as median and IQR, categorical variables are presented as frequency and percentage. For group comparisons, the Mann–Whitney *U*-test was used to determine differences in medians. Non-parametric between-group-comparisons were performed with Pearson’s Chi-square test

**Table 3:** Group comparison according to median serum levels of complement C3

	C3 < 1.3 g/L	C3 ≥ 1.3 g/L	*p* value
Serum C3–g/L	1.02 (0.81–1.20)	1.41 (1.32–1.55)	< 0.0001
Serum C4–g/L	0.22 (0.15–0.31)	0.29 (0.22–0.30)	0.0706
Serum creatinine–mg/dL	4.72 (1.75–7.27)	2.91 (0.87–4.14)	0.0088
eGFR–mL/min/1.73 m^2^	9.80 (6.90–27.90)	24.10 (13.20–89.90)	0.0121
CRP–mg/L	57.40 (10.80–89.00)	63.80 (30.30–109.30)	0.3432
Female sex–no. (%)	8 (42.11)	9 (47.37)	0.7442
Age–years	69 (55–76)	60 (50–70)	0.1882
ANCA diagnosis			
MPA–no. (%)	12 (63.2)	10 (52.63)	
GPA–no. (%)	7 (36.8)	9 (47.37)	0.5111
ANCA subtype			
MPO–no. (%)	12 (63.16)	10 (52.63)	
PR3–no. (%)	7 (36.84)	9 (47.37)	0.5111
Relapse–no. (%)	4 (21.05)	2 (10.53)	0.3736
BVAS–points	18 (15.00–20.00)	18 (15–21)	0.9937
KRT within 40 days–no. (%)	10 (52.63)	3 (15.79)	0.0167
Death within 40 days–no. (%)	1 (5.26)	0 (0.00)	0.3109
Berden class			
Sclerotic class–no. (%)	2 (10.53)	0 (0.00)	
Crescentic class–no. (%)	7 (36.84)	7 (36.84)	
Focal class–no. (%)	8 (42.11)	11 (57.89)	
Mixed class–no. (%)	2 (10.53)	1 (5.26)	0.4223
ANCA Renal Risk Score			
High risk–no. (%)	5 (26.31)	1 (5.26)	
Intermediate risk–no. (%)	7 (36.84)	9 (47.37)	
Low risk–no. (%)	7 (36.84)	9 (47.37)	0.2053
Histopathological lesions			
Normal glomeruli–% of total	42.42 (11.11–70.59)	56.25 (35.29–83.33)	0.1280
Crescentic glomeruli–% of total	37.50 (12.50–54.55)	34.38 (0.00–60.71)	0.5475
Necrotic glomeruli–% of total	15.15 (0.00–46.67)	28.00 (9.09–51.06)	0.3701
Sclerotic glomeruli–% of total	9.09 (0.00–29.41)	0.00 (0.00–12.50)	0.0646
IF/TA–%	20.00 (15.00–40.00)	20.00 (5.00–40.00)	0.3246

*ANCA* anti-neutrophil cytoplasmic antibody, *BVAS* Birmingham Vasculitis Activity Score, *CRP* C-reactive protein, *eGFR* estimated glomerular filtration rate (CKD-EPI), *GPA* granulomatosis with polyangiitis, *IF/TA* interstitial fibrosis/tubular atrophy, *IQR* interquartile range, *KRT* kidney replacement therapy, *MPA* microscopic polyangiitis, *MPO* myeloperoxidase, *no.* number, *PR3* proteinase 3 Continuous variables are expressed as median and IQR, categorical variables are presented as frequency and percentage. For group comparisons, the Mann–Whitney *U*-test was used to determine differences in medians. Non-parametric between-group-comparisons were performed with Pearson’s Chi-square test

## Discussion

Low serum complement C3 levels without hypocomplementemia per se are an independent predictor of poor renal prognosis in patients with ANCA-associated renal vasculitis [2]. We performed a comparative analysis with regard to serum C3 and C4 levels to test its clinical implications in ANCA-associated renal vasculitis. As observed for levels of C3, we identified C4 hypocomplementemia only in a minor subset. As previously described, we defined low serum C4 according to median levels and identified no association with risk of KRT requirement or death in ANCA-associated renal vasculitis [2]. While most cases of ANCA-associated renal vasculitis showed an overlap of low complement C3 and C4 levels, there were subsets of patients with isolated C4 lowering. As compared to previously described prediction of poor outcome by low levels of complement C3, low levels of complement C4 did not associate with KRT or death. Interestingly, a minor fraction that experienced KRT or death had isolated C4 lowering not captured by serum C3 measurements. These observations implicate overlapping, but also independent roles of complement C3 and C4 that might contribute to kidney injury in ANCA-associated renal vasculitis. This is in line with our previous observation in this cohort that low levels of either serum C3 or C4 correlated with distinct inflammatory lesions in ANCA-associated renal vasculitis [10]. Regarding potential clinical implications, low serum levels of C3 were associated with renal injury as previously and independently described [2–5]. In contrast, low levels of complement C4 were associated with older age in ANCA-associated renal vasculitis independent of kidney function, ANCA subtype, relapsing disease, systemic vasculitis activity, or classification of ANCA-associated renal vasculitis. While a relationship between age and distinct complement components has already been described in studies of healthy populations, this has not yet been observed in ANCA-associated renal vasculitis [15]. Its relevance related to age and treatment response remains elusive and requires further investigation. Currently, two C5a inhibitors are in clinical development for AAV: the oral C5a receptor (C5aR) inhibitor avacopan and the monoclonal C5a antibody IFX-1 [16]. Safety and efficacy with steroid-sparing effects of avacopan in patients with GPA/MPA have already been shown [16]. Furthermore, IFX-1 has entered Phase II development [16]. Therefore, detailed knowledge of distinct complement components contributing to kidney injury could be of relevance to further improve current strategies targeting the complement system, which is especially relevant in severe ANCA-associated renal vasculitis [17, 18].

The main limitations of our study are its retrospective design and the small patient number. Furthermore, we only assessed outcome during the initial course of ANCA-associated renal vasculitis requiring validation in independent cohorts also regarding long-term renal and overall outcome. Nevertheless, our observation that a minor fraction with isolated C4 lowering was not captured by serum C3 measurements implies overlapping, but also independent roles of complement C3 and C4 in ANCA-associated renal vasculitis. Therefore, a detailed analysis of intrarenal complement C4 deposition in comparison with C3 deposits among other histopathological lesions would be of relevance to gain further insights into the role of distinct complement components in ANCA-associated renal vasculitis.

## Data Availability

Deidentified data are available on reasonable request from the corresponding author.

## References

[1] Riedl M (2017). Complement activation induces neutrophil adhesion and neutrophil-platelet aggregate formation on vascular endothelial cells. Kidney Int Rep.

[2] Augusto JF (2016). Low serum complement C3 levels at diagnosis of renal ANCA-associated vasculitis is associated with poor prognosis. PLoS ONE.

[3] Tampe D (2022). Low serum levels of complement C3c at diagnosis indicate poor outcome in antineutrophil cytoplasmic antibody-associated glomerulonephritis. Kidney Int Rep.

[4] Crnogorac M (2018). Serum C3 complement levels in ANCA associated vasculitis at diagnosis is a predictor of patient and renal outcome. J Nephrol.

[5] Lionaki S (2021). Hypocomplementemia at diagnosis of pauci-immune glomerulonephritis is associated with advanced histopathological activity index and high probability of treatment resistance. Kidney Int Rep.

[6] Hakroush S (2020). Histopathological findings predict renal recovery in severe ANCA-associated vasculitis requiring intensive care treatment. Front Med (Lausanne).

[7] Hakroush S (2021). Systematic histological scoring reveals more prominent interstitial inflammation in myeloperoxidase-ANCA compared to proteinase 3-ANCA glomerulonephritis. J Clin Med.

[8] Tampe D (2021). Proteinuria indicates decreased normal glomeruli in ANCA-associated glomerulonephritis independent of systemic disease activity. J Clin Med.

[9] Hakroush S (2020). Variable expression of programmed cell death protein 1-ligand 1 in kidneys independent of immune checkpoint inhibition. Front Immunol.

[10] Hakroush S (2021). Complement components C3 and C4 indicate vasculitis manifestations to distinct renal compartments in ANCA-associated glomerulonephritis. Int J Mol Sci.

[11] Mukhtyar C (2009). Modification and validation of the Birmingham Vasculitis Activity Score (version 3). Ann Rheum Dis.

[12] Levey AS (2009). A new equation to estimate glomerular filtration rate. Ann Intern Med.

[13] Berden AE (2010). Histopathologic classification of ANCA-associated glomerulonephritis. J Am Soc Nephrol.

[14] Brix SR (2018). Development and validation of a renal risk score in ANCA-associated glomerulonephritis. Kidney Int.

[15] Gaya da Costa M (2018). Age and sex-associated changes of complement activity and complement levels in a healthy caucasian population. Front Immunol.

[16] Jayne D (2019). Complement inhibition in ANCA vasculitis. Nephrol Ther.

[17] Brilland B (2021). Low complement C3 levels at diagnosis of ANCA-associated glomerulonephritis, a specific subset of patients to target with Anti-C5aR therapy? In response to: hypocomplementemia at diagnosis of pauci-immune glomerulonephritis is associated with advanced histopathological activity index and high probability of treatment resistance. Kidney Int Rep.

[18] Lionaki S, Boletis JN (2021). Low serum C3 pauci-immune glomerulonephritis: high histopathological activity and lower rates of response to standard therapies. Kidney Int Rep.

